# Estimation versus measurement of the glomerular filtration rate for kidney function assessment in patients with cancer undergoing cisplatin-based chemotherapy

**DOI:** 10.1038/s41598-020-68010-5

**Published:** 2020-07-08

**Authors:** Marie-Christin Klöckl, Anne-Katrin Kasparek, Jakob M. Riedl, Florian Moik, Stefanie Mollnar, Michael Stotz, Joanna Szkandera, Angelika Terbuch, Armin Gerger, Tobias Niedrist, Martin Pichler, Thomas Bauernhofer, Gernot Schilcher, Sabine Zitta, Alexander R. Rosenkranz, Claudia Friedl, Herbert Stöger, Florian Posch

**Affiliations:** 1 Division of Oncology, Department of Internal Medicine, Comprehensive Cancer Center Graz, Medical University of Graz, Auenbruggerplatz 15, 8036 Graz, Austria; 2 Center for Biomarker Research in Medicine (CBmed), Graz, Austria; 3 Research Unit “Genetic Epidemiology and Pharmacogenetics”, Medical University of Graz, Graz, Austria; 4 Clinical Institute of Medical and Chemical Laboratory Diagnostics, Medical University of Graz, Graz, Austria; 5 Research Unit “Non-Coding RNAs and Genome Editing in Cancer”, Medical University of Graz, Graz, Austria; 6 Department of Experimental Therapeutics, University of Texas M.D. Anderson Cancer Center, Houston, TX USA; 7 Division of Nephrology, Department of Internal Medicine, Medical University of Graz, Graz, Austria

**Keywords:** Acute kidney injury, Chemotherapy

## Abstract

Glomerular filtration rate (GFR) assessment is indicated before every administration of cisplatin. The optimal modality for this purpose [GFR measurement by urinary Creatinine Clearance (uCrCl) versus GFR estimation (eGFR) by the CKD-EPI formula versus both] is unclear. We investigated whether eGFR only is safe in this setting. Paired uCrCl and eGFR determinations from 470 cisplatin cycles from 121 patients were analyzed [median age: 55 years; most frequent tumor site: genitourinary (45%); palliative treatment: n = 41 (34%)]. Primary endpoint was the proportion of cycles with uCrCl < 50 ml/min/1.73m^2^ and eGFR ≥ 50 ml/min/1.73m^2^ (i.e. a “false negative” result when only determining eGFR). The primary endpoint occurred in 8 of 470 cisplatin cycles (1.7%, 95%CI 0.5–2.9). In all 8 events, uCrCl was lower than eGFR (mean uCrCl vs. eGFR: 43 versus 112 ml/min/1.73m^2^). The uCrCl was re-measured in all patients, and showed normal results in all but 1 patient. None of these events precluded the administration of cisplatin at the planned date, and no subsequent cases of acute nephrotoxicity occurred. Overall agreement between uCrCl and eGFR was low, with qualitative analysis suggesting frequent incompliance with 24-h urine collection. We conclude that an eGFR is sufficient for assessing kidney function in patients with cancer undergoing cisplatin therapy.

## Introduction

Cisplatin is a widely-used antineoplastic drug that is essential for the therapy of a broad range of solid and hematologic cancers in children^1^, adolescents^2^, and adults^3,4^. Acute kidney injury (AKI) is a rare but potentially life-threatening complication of cisplatin therapy^5^. Because this complication occurs predominantly in patients with already pre-existing kidney dysfunction, cisplatin is widely considered to be contraindicated in patients with a glomerular filtration rate (GFR) below 50–60 ml/min/1.73m^2^^6,7^. To identify these patients, a pre-treatment evaluation of kidney function by determining the GFR is mandatory in every patient before every cisplatin administration^8^. However, it is unclear which method of GFR assessment is optimal for this purpose, with some centers opting for (1) a GFR *measurement* by urinary Creatinine Clearance (uCrCl), (2) an *estimated* GFR by a validated formula such as CKD-EPI (eGFR), or (3) both^9^. The uCrCl has the advantage of being the potentially most accurate routinely-available method of GFR assessment (“gold standard”, ignoring non-routinely available methods of GFR measurement such as inulin or iothalamate clearance or nuclear medicine techniques measuring plasma clearance of the radio-isotope Technetium-99 m-diethyl-triamine-penta-acetic acid (Tc-99 m-DTPA))^10^, but requires a timed (mostly 24-h) urine collection which is burdensome for patients. Moreover, erroneous uCrCl determinants resulting from compliance problems with timed 24-h urine collection on the patient side can result in wrong uCrCl values necessitating repetition of urine collection and thus extra inpatient days, inconvenience for patients and healthcare providers, additional costs and a delay in cisplatin administration^11^. In contrast, the eGFR is based on validated estimation formulae such as the Chronic Kidney Disease Epidemiology Collaboration (CKD-EPI) formula and can be assessed with a single blood draw at the day of cisplatin administration^12,13^. Despite this conceptual advantage of simplicity, it has not yet been investigated whether a strategy of determining the eGFR only before cisplatin therapy (i.e. omitting uCrCl measurement) is safe for patients by preventing acute kidney injury. Thus, in this retrospective study, we investigated the agreement between these two methods in patients with cancer undergoing cisplatin chemotherapy, with the ultimate aim of evaluating whether uCrCl measurement can be safely omitted in pre-cisplatin kidney function assessment.

## Methods

### Study design and population

This study was a retrospective, single-center, observational cohort study including adult patients (i.e. ≥ 18 years) with histologically-confirmed solid cancer who had received at least one dose of cisplatin (± other agents) at the Division of Oncology, Department of Internal Medicine, Medical University of Graz, Austria, between Jan 1st, 2015, and Apr, 4th, 2016. These criteria led to the identification of 127 patients, of whom 6 patients (5%) were excluded because they eventually did not receive cisplatin (n = 1 due to compliance problems, n = 1 because cisplatin was already initiated earlier at an extramural facility, and n = 4 because kidney function declined below the local threshold in the interval between cisplatin indication and first treatment cycle). Thus, 121 patients could be included in the study. Clinical and laboratory data were retrieved retrospectively from our prospectively-maintained in-house electronic health care database as previously described.^14–19^ The study was conducted in accordance with the Declaration of Helsinki, and the local institutional review board approved all aspects of the study protocol (Ethics Committee of the Medical University of Graz, Approval number: EK 29–596 ex 16/17, ethikkommission@medunigraz.at).

### Local cisplatin indication protocol before and during therapy according to renal function

The eligibility for cisplatin in light of renal function was continuously (re-)evaluated by the treating oncologist before and during cisplatin treatment. Consistent with best-practice recommendations, every patient received forced hydration before, during and after the application of cisplatin^8,20^. The specific cisplatin-dose-adapted hydration schedules remained the same over the study period, with an example being reported in Supplementary Table 1. Patients with uCrCl and/or eGFR < 50 ml/min/1.73 m^2^ at the time of therapy evaluation by an oncologist were considered ineligible for cisplatin therapy (contraindication against cisplatin according to the local drug label). Patients with uCrCl and/or eGFR ≥ 60 ml/min/1.73 m^2^ at the time of therapy evaluation by an oncologist were considered to have no nephrologic contraindication against cisplatin. Patients with uCrCl and/or eGFR between 50–60 ml/min/1.73 m^2^ could receive cisplatin on a “split-dose” schedule^21^, which means the cumulative dose of cisplatin is applied on 2 days at least 1 week apart. Patients with a new-onset impairment of kidney function during cisplatin therapy (decline in uCrCl and/or eGFR < 50 ml/min/1.73 m^2^) did not receive cisplatin anymore. They were switched to carboplatin or to any other cisplatin-free regimen available in the pertinent indication.

**Table 1:** Baseline characteristics of the study population. Continuous variables are summarized as medians [25th percentile (Q1)—75th percentile (Q3)], whereas categorical variables are reported as absolute frequencies and percentages. n (% miss.) reports the number of fully observed patients (% with missing data)

Variable	N (% miss.)	Summary estimate (median [25th–75th percentile] or absolute frequency (%))
**Demographics**		
Age at cisplatin initiation (years)	121 (0%)	55.2 [41.6–63.0]
Female Gender	121 (0%)	33 (27%)
Body Mass Index (BMI) at cisplatin initiation (kg/m^2^)	121 (0%)	24.7 [22.7–27.1]
**Primary tumor sites**	121 (0%)	–
Genitourinary	–	54 (45%)
Upper gastrointestinal tract	–	31 (26%)
Thoracic	–	25 (21%)
Cancer of unknown primary (CUP)	–	4 (3%)
Breast	–	2 (2%)
Head and neck	–	2 (2%)
Lower gastrointestinal tract	–	2 (2%)
Other	–	1 (1%)
**Treatment strategy**	121 (0%)	–
Palliative	–	41 (34%)
Curative^a^	–	26 (22%)
Neoadjuvant	–	26 (22%)
Adjuvant	–	26 (22%)
Pseudoadjuvant	–	2 (2%)
**Cisplatin chemotherapy schemes**	121 (0%)	–
Cisplatin monotherapy		4 (3%)
Cisplatin/5-FU (± targeted agent)		18 (15%)
Cisplatin/pemetrexed		8 (7%)
Cisplatin/etoposide		19 (16%)
Cisplatin/etoposid/bleomycin (“PEB”)		28 (23%)
Cisplatin/vinorelbine		8 (7%)
Cisplatin/gemcitabine		28 (23%)
Cisplatin/capecitabine (± targeted agent)		4 (3%)
Others		4 (3%)
**Projected cisplatin treatment cycles**	121 (0%)	–
1–2	–	21 (17%)
3–4	–	76 (63%)
5–6	–	10 (8%)
Not specified before start of therapy	–	14 (12%)
**Cisplatin therapy data (n = 480 cycles)**		
Number of received cisplatin cycles per patient	121 (0%)	4 [3–5]
Cumulative received cisplatin dose per cycle (mg/m^2^ BSA)	480 (0%)	75 [75–100]
Cumulative received cisplatin dose per patient (mg/m^2^ BSA)	121 (0%)	300 [225–400]
**Cisplatin therapy delay data**		
Number of cisplatin cycles delayed for at least one day	470 (2%)	116 (25%)
Time of delay (days)	116 (0%)	6 [2–8]
**Reasons for delay**	79 (32%)	–
Infection or suspected infection	–	16
Neutropenia	–	14
Other	–	13
Port-A-Cath implantation	–	8
Change in regimen timing	–	7
Hematotoxicity	–	6
Febrile neutropenia	–	5
Restaging	–	5
Venous thromboembolism	–	3
Dental extraction	–	2

^a^ “Curative” means that chemotherapy was administered as sole therapy in a curative intent, i.e. this applies to patients with e.g. advanced testicular germ cell tumors, and patients with other “curative” treatment strategies (e.g. neoadjuvant or adjuvant) are not listed in this category.

*5-FU* 5-fluorouracil, *BSA* body surface area.

### Glomerular filtration rate (GFR) estimation and measurement protocol

According to local standard, all patients collected urine for 24 h starting the day before the projected start of each cisplatin cycle. At the first day of each cycle, patients are admitted to the inpatient ward of our department, and venous blood is collected by sterile antecubital venipuncture or from a venous Port-A-Cath system. Then, blood samples drawn into an 8 ml lithium heparin tube with separation gel (Greiner Bio-One Vacuette, Austria) and the 24-h urine were sent to the local laboratory for determination of creatinine. In both blood plasma and urine, our laboratory used an ID/MS standardized, automated assay of Jaffe’s reaction on the cobas® 8,000 c502 analyzer by Roche Diagnostics (“CREJ2”). The eGFR was estimated in all patients with the CKD-EPI formula^12^. The uCrCl was calculated in all patients using the following formula, where BSA is the body surface area according to the Du Bois formula and 1.73 is the average human BSA:^22^

$$uCrCl = \frac{{creatinine_{{urine}} }}{{creatinine_{{plasma}} }} \times \frac{{volume_{{urine}} }}{{time_{{sampling}} }} \div BSA \times 1.73$$

### Endpoints and sensitivity analysis

The primary endpoint of this study was the proportion of cycles where the uCrCl was < 50 ml/min/1.73m^2^ while the eGFR was ≥ 50 ml/min/1.73m^2^ (i.e. the proportion of cycles where forgoing uCrCl measurement would lead to a “false-negative” test result in terms of kidney function assessment prior cisplatin therapy). Secondary endpoint was the agreement between the uCrCl and the eGFR for kidney function assessment. This endpoint was expressed as (1) an absolute difference, (2) an intra-class correlation^23^, and (3) modified limits of agreement (LoA) accounting for the clustered nature of the data^24^. Tertiary endpoints included: (1) the longitudinal change in the eGFR during therapy^25^, and (2) the risk of developing an at least 30% relative decline in the pre-cisplatin-treatment eGFR during cisplatin therapy^26^. In a sensitivity analysis, we repeated the primary endpoint analysis by excluding samples with potentially incomplete 24-h urine collection, as indicated by a urine creatinine/body weight ratio (in mmol/kg/24-h) lower than age-, race-, and sex-adapted thresholds at the 10^th^ percentile of the ratio distribution in patients with CKD stages 1–5.^27^.

### Statistical methods

All statistical analyses were performed using Stata (Windows version 15.0, Stata Corp., Houston, TX, USA) and MedCalc (Windows version 18.5, MedCalc Software bvba, Ostend, Belgium). Continuous variables were reported as medians [25th–75th percentile], whereas count data were reported as absolute frequencies (%). The primary endpoint was expressed as a proportion with 95% binomial exact confidence intervals. The secondary endpoint was expressed as a mean difference on an absolute scale (in ml/min/1.73m^2^) as well as an intra-class correlation coefficient (iCCC). The first two secondary endpoints were obtained from a linear mixed random-intercept model accounting for the clustered nature of kidney function measurement within individual patients (Stata routine mixed)^28^. The third secondary endpoint was obtained using a modified Bland–Altman analysis in MedCalc accounting for the clustered structure of data (i.e. one or more eGFR measurements contributed by a single individual patient)^24^. The longitudinal change in the eGFR during therapy was estimated as a change in ml/min/1.73m^2^/month using a linear mixed growth model with a random-intercept-and-slope model (Stata routine mixed)^25,28^, while the risk of developing a 30% relative decline in eGFR over time was estimated with a 1-Kaplan–Meier estimator.

### Ethical approval

All procedures performed in studies involving human participants were in accordance with the ethical standards of the institutional and/or national research committee and with the 1964 Helsinki declaration and its later amendments or comparable ethical standards. As this is a retrospective study, formal consent from individual patients was not required. This “waiver of consent” was approved by the local institutional review board (Ethics Committee of the Medical University of Graz, Approval number: EK 29–596 ex 16/17, ethikkommission@medunigraz.at).

## Results

### Study cohort and treatment data

One-hundred-and-twenty-one patients were included in the analysis (Table 1). At cisplatin initiation, the median age of the cohort was 55 years, and 33 patients (27%) were female. The most frequent primary tumor sites were genitourinary (45%), upper gastrointestinal tract (26%), and thorax (21%). Treatment intent was palliative in 41 patients (34%), and the vast majority of patients (n = 117, 97%) received cisplatin-based combination therapy. Only four patients (3%) received cisplatin monotherapy.

### Kidney function measurement before and during cisplatin therapy

Overall, the 121 patients received a total of 480 treatment cycles with cisplatin (Table 1). Kidney function was assessed with the eGFR prior to all 480 cycles, and with a concurrent uCrCl measurement in 470 cycles. The 10 uCrCl measurements were missing either due to incompliance with the local protocol on the institutional side (n = 6 measurements) or due to acute chemotherapy initiation in a young and severely-ill patient with newly-diagnosed diffusely-metastatic germ cell tumor at the intensive care unit (n = 4 measurements from 4 cycles in the same patient, Supplementary Fig. 1). Overall, the median eGFR and uCrCl values at cisplatin initiation were 94 ml/min/1.73m^2^ and 102 ml/min/1.73m^2^, respectively (Table 2).

**Figure 1:**
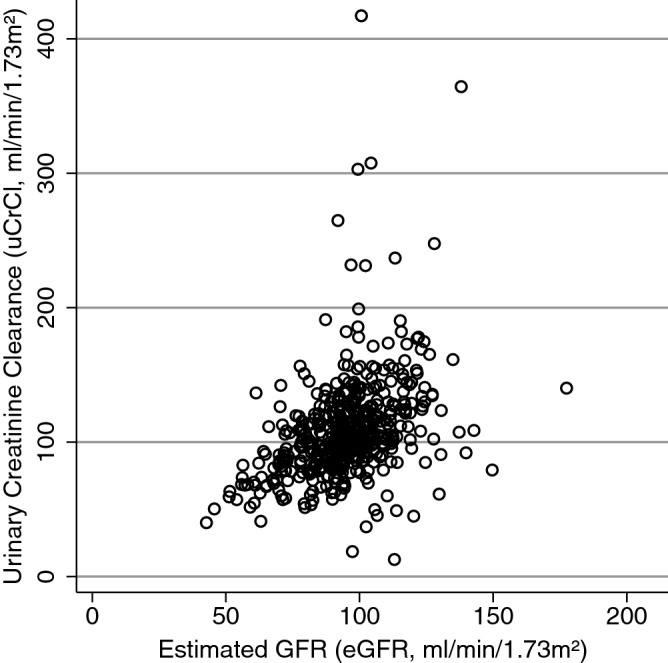
Secondary endpoint: agreement between uCrCl and eGFR—scatter plot. Every hollow circle represents a single paired eGFR/uCrCl measurement. Strong variability in uCrCl is observed, with some patients having highly outlying uCrCl measurements. In contrast, the variability of eGFR determinations is much smaller. *eGFR* estimated glomerular filtration rate, *uCrCl* urinary creatinine clearance.

**Table 2:** Comparison of uCrCl and eGFR data at cisplatin initiation, before each cisplatin cycle, and its change during therapy. Reported results are medians [25th–75th percentile], ranges [5th–95th percentile], and changes in kidney function over time during therapy (in ml/min/1.73m^2^/month. Changes were estimated with a linear mixed model taking into account the clustered nature of the data (Stata routine mixed).

Variable	eGFR (CKD-EPI, ml/min/1.73m^2^)	Urinary Creatinine Clearance (uCrCl, ml/min/1.73m^2^)
**At Cisplatin initiation (n = 121)**		
Kidney function (median [25th–75th percentile]	94 [82–106]	102 [84–119]
Kidney function (range [5th–95th percentile])	43–135 [62–121]	40–303 [57–155]
**Before each cycle (n = 480)**		
Kidney function (median [25th–75th percentile]	95 [85–105]	104 [87–124]
Kidney function (range [5th–95th percentile])	43–189 [67–124]	13–417 [61–165]
**Change during therapy**		
in ml/min/1.73m^2^/month (95%CI, *p*)	− 0.1 (− 0.5–0.3, *p* = 0.568)	− 0.5 (− 1.8–0.8, *p* = 0.425)

*eGFR* estimated glomerular filtration rate, *CKD-EPI* chronic kidney disease epidemiology collaboration equation, *uCrCl* urinary creatinine clearance, *95% CI* 95% confidence interval, *p* Wald test *p* value.

### Primary endpoint: proportion of “false-negative” kidney function assessments upon forgoing CrCl measurement

The primary endpoint occurred in 8 out of 470 cycles (1.7%, 95%CI 0.5–2.9, *p* = 0.004). These 8 primary endpoint events were contributed by 7 individual patients (Table 3). In all of these 8 events (from 7 individual patients), the uCrCl was lower than the eGFR (mean uCrCl vs. eGFR: 43 ml/min/1.73m^2^ vs. 112 ml/min/1.73m^2^, mean difference: 65 ml/min/1.73m^2^ (95%CI 50–80, *p* < 0.0001), range 22–101 ml/min/1.73m^2^). Qualitative analysis of hospital records showed that none of the 8 events led to a delay in chemotherapy administration (Table 3). In detail, uCrCl was re-measured the next day and yielded normal results in 3 cases (suspected incompliance with outpatient 24-h urine collection) and an abnormal result in 1 case (suspected incompliance with inpatient 24-h urine collection). Urinary creatinine Clearance was 49.1 ml/min/1.73m^2^ and 49.8 ml/min/1.73m^2^ (i.e. very close to the local cisplatin indication threshold at 50 ml/min/1.73m^2^) in 2 cases (and chemotherapy was given regularly by treating physicians without uCrCl re-measurement by rounding up uCrCl to 50 ml/min/1.73m^2^, thus violating the local protocol). In one patient chemotherapy was given despite an uCrCl measurement of 41 ml/min/1.73m^2^ due to vital indication (extensive retroperitoneal germ cell tumor with inferior-vena-cava syndrome).

**Table 3:** Descriptive and qualitative analysis of primary endpoint events.

Patient	Diagnosis	Age (years)	Cycle #	Height (cm) /weight (kg) /Body Surface Area (m^2^)	eGFR (CKD-EPI, ml/min/1.73m^2^)	Urinary Creatinine Clearance (uCrCl, ml/min/1.73m^2^)	Outcome	Chemotherapy administered without delay?
#1	Testicular cancer	26	4	180 cm 69 kg 1.87m^2^	120	45	uCrCl was repeated the next day and was 118 ml/min/1.73m^2^	Yes
#2	Testicular cancer	47	3	178 cm 73 kg 1.90m^2^	102	37	uCrCl was repeated the next day and was 104 ml/min/1.73m^2^	Yes
#3	Biliary tract cancer	68	4	170 cm 73 kg 1.84m^2^	97	19	Suspected incompliance with 24-h urine collection, uCrCl was repeated the next day and was 79 ml/min/1.73m^2^	Yes
#4	Testicular cancer	37	1	171 cm 65 kg 1.76m^2^	63	41	Chemotherapy was administered despite uCrCl result of 41 because of vital indication (extensive metastatic disease)	Yes
#5	Biliary tract cancer	69	2	159 cm 47 kg 1.45m^2^	105	50 (49.8)	Result was rounded to 50 by treating physician	Yes
#6	Biliary tract cancer	36	1	180 cm 84 kg 2.04m^2^	107	46	Suspected incompliance with 24-h urine collection, uCrCl was repeated 8 days later and was 69 ml/min/1.73m^2^	Yes
#6	Biliary tract cancer	36	3	180 cm 84 kg 2.04m^2^	113	13	Suspected incompliance with 24-h urine collection, uCrCl was repeated 14 days later and was 93 ml/min/1.73m^2^	Yes
#7	CUP syndrome	48	4	193 cm 78 kg 2.08m^2^	114	50 (49.1)	Suspected incompliance with 24-h urine collection, uCrCl was repeated the next day and was 37 ml/min/1.73m^2^ (Patient incompliant with 24-h urine collection also in inpatient setting)	Yes

*eGFR* estimated glomerular filtration rate, *CKD-EPI* chronic kidney disease epidemiology collaboration equation, *uCrCl* urinary creatinine clearance, *CUP* cancer of unknown primary.

### Secondary endpoint: agreement between eGFR and CrCl before and during cisplatin therapy

**Figure 2:**
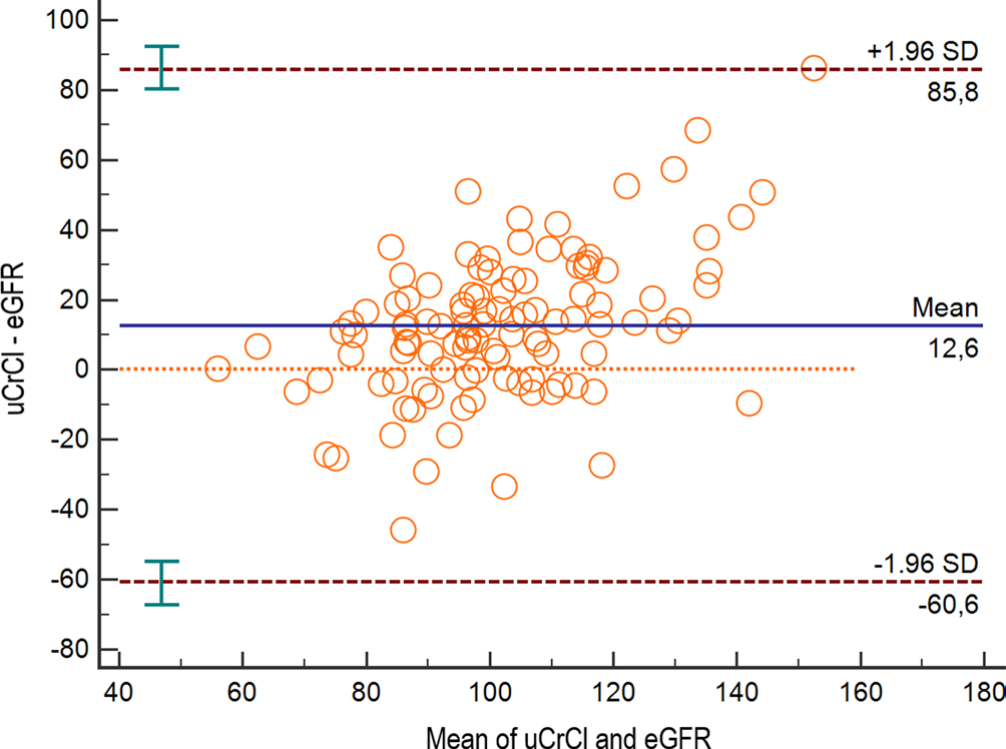
Secondary endpoint: agreement between uCrCl and eGFR—modified bland–altman analysis. This modified analysis specifically takes into account the clustered nature of the data, i.e. that individual patients contribute more than one paired uCrCl/eGFR reading. Each hollow circle represents the paired within-patient eGFR/uCrCl measurements that were averaged within a patient, i.e. each hollow circle represents a single patient. Patients with only 1 paired measurement (i.e. only 1 cycle) were excluded from this analysis. The blue solid horizontal line represents the mean within-patient-averaged difference between uCrCl and eGFR. As this is above the orange dashed line of zero difference, uCrCl measurements are systematically higher than eGFR measurements in our study population. The dashed horizontal red lines represent the 95% limits of agreement (LoA). *SD* standard deviation, *uCrCl* urinary creatinine clearance, *eGFR* estimated glomerular filtration rate.

In the overall population, uCrCl measurements were significantly higher than eGFR estimates (mean difference from linear mixed model = 13.0 ml/min/1.73m^2^, 95%CI 9.0–16.9, *p* < 0.0001). The eGFR and the uCrCl were in adequate agreement for most patients, however, several highly outlying uCrCl values were observed, with outliers both on the low and high end of kidney function (Fig. 1). These outliers led to a relatively low overall agreement between the two methods for kidney function assessment (Intra-Class Coefficient from linear mixed model = 0.72, 95%CI 0.64–0.78). In a modified Bland–Altman analysis of agreement accounting for the clustered nature of the data, the 95% limits of agreement between eGFR and uCrCl were − 61 to 86 (Fig. 2).

### Tertiary endpoint: longitudinal change in kidney function during cisplatin therapy

On average, neither eGFR nor uCrCl significantly changed during cisplatin therapy (Table 2). Also the difference between eGFR and uCrCl did not change over time (change =  − 0.3 ml/min/1.73m^2^/month, 95%CI − 1.5–0.85, *p* = 0.576).

In a further endpoint analysis, two individual patients (1.7%, 95%CI 0.2–5.8) developed a relative decline in eGFR ≥ 30% (as compared to the eGFR prior the 1st cycle) at least once over time. The first patient had a baseline eGFR of 80 ml/min/1.73m^2^ and declined to 56 ml/min/1.73m^2^ at the sixth (last) treatment cycle. The second patient had a baseline eGFR of 85 ml/min/1.73m^2^ and declined to 58 ml/min/1.73m^2^ also at the sixth (last) treatment cycle.

In contrast, 30 patients (25%, 95%CI 17–33) developed a relative decline in uCrCl ≥ 30% (as compared to the uCrCl prior the 1^st^ cycle) at least once over time. In these 30 patients the median uCrCl at the first cycle was 116 ml/min/1.73m^2^ [25th–75th percentile: 103–149, range 46–303]. The declines occurred in 65 individual cycles, and the median uCrCl in cycles with at least 30% decline was 83 ml/min/1.73m^2^ [71–105, range 13–169].

As declining muscle mass due to cancer cachexia and chemotherapy side effects may lead to decreased serum creatinine and thus falsely high eGFR, we analyzed weight changes (as a proxy for muscle mass) during chemotherapy. During a median of 4 cycles of cisplatin therapy [25th–75th percentile: 3–5, Table 1], average patient weight declined by 0.4 kg per treatment cycle (change/cycle =  − 0.37 kg, 95%CI − 0.62–(− 0.12), *p* = 0.004).

### Sensitivity analysis: excluding samples with potentially incomplete urine collection

Using thresholds for “complete” 24-h urine collection from a previously-published renal physiology study (as defined by a ratio of 24-h urine creatinine to body weight (in mmol/kg/24 h) < 10th percentile of age-, race-, and sex-adapted values)^27^, we found that 24-h urine collection may have been incomplete in 178 (38%) of the 470 chemotherapy cycles that were analyzed. In these 178 cycles, the median difference [25th–75th percentile] between these ratios and their thresholds was − 0.02 [− 0.01–(− 0.04)]. Upon excluding these 178 cycles, the primary endpoint occurred in only one (0.3%) out of 292 chemotherapy cycles.

## Discussion

In this retrospective study comparing *measurement* versus *estimation* of GFR in patients with cancer undergoing chemotherapy with cisplatin we showed that a strategy of eGFR determination only is a safe way to assess kidney function. Thus, routine *measurement* of the uCrCl can be safely omitted in favor of routine GFR *estimation* in patients with cancer who have received at least one dose of cisplatin. This finding simplifies supportive care for kidney protection in the global population of patients requiring treatment with cisplatin.

Assessment of kidney function is indicated in all patients undergoing cisplatin therapy at treatment initiation and at each subsequent cisplatin cycle^29^. Our study was motivated by the fact that considerable uncertainty existed on the optimal modality of kidney function assessment in this setting^9^. For patients, a simple blood draw at the day or the day before cisplatin therapy to obtain the eGFR based on the serum creatinine level, age, race, and sex (CKD-EPI formula) is obviously much more convenient than a 24-h urine collection protocol that is required for uCrCl measurement^12,30^. Nonetheless, the more burdensome uCrCl offers at least the potential of being more accurate and thus potentially enabling oncologists to discover more patients with impaired kidney function who are at risk for cisplatin-induced nephrotoxicity. Given our institutional policy of measuring both parameters in all patients undergoing cisplatin therapy before each cycle, we could include paired within-patient eGFR and uCrCl data from 470 cisplatin cycles from 121 all-comer patients with solid tumors into this retrospective observational study, and address whether it may be safe to omit uCrCl measurement at all.

To answer this question, we defined an endpoint that takes into account the widely-considered contraindication against cisplatin administration at eGFRs or uCrCls < 50 ml/min/1.73m^2^ as well as the potential discordance between eGFR and uCrCl results. Should a patient have eGFR and uCrCl measurements < 50 ml/min/1.73m^2^, a strategy of omitting uCrCl measurement would not have resulted in information loss. Similarly, no information loss with omitting uCrCl measurement would occur in a situation when eGFR and uCrCl are both ≥ 50 ml/min/1.73m^2^, or when the eGFR is < 50 ml/min/1.73m^2^ while the uCrCl is ≥ 50 ml/min/1.73m^2^. However, potential for harming the patient by cisplatin administration without prior uCrCl measurements exists in a situation where the eGFR is ≥ 50 ml/min/1.73m^2^ while the true uCrCl would be < 50 ml/min/1.73m^2^. Thus, only the last scenario is relevant for assessing whether it is safe to omit uCrCl measurement prior cisplatin therapy. This scenario was thus framed as the primary endpoint of the study, and occurred in 8 out of 480 cycles from 7 out of 121 patients. Given the large adverse impact that a cisplatin-induced kidney injury can have on patient outcome, this proportion appears to be too high for omitting uCrCl at first. However, qualitative analysis of patient charts showed that the low uCrCl readings in these 7 patients were mostly due to incompliance/technical problems with 24-h urine collection on the patient side, i.e. they were not indicative of true kidney dysfunction. Indeed, uCrCl was normal in 5 out of 8 cycles after immediate uCrCl re-measurement within an inpatient setting. In 2 more cycles uCrCl was between 49 ml/min/1.73m^2^ and 50 ml/min/1.73m^2^ upon inpatient re-measurement, and only truly below 50 ml/min/1.73m^2^ upon inpatient re-measurement in one cycle. Importantly, none of these uCrCl measurements resulted in patient delay, as cisplatin was administered at the planned time in the 5 cycles with normalized uCrCl upon re-measurement as well as the 2 cycles with borderline uCrCl upon re-measurement. In the patient with truly impaired uCrCl (41 ml/min/1.73m^2^ subsequent to incipient hydronephrosis due to bulky retroperitoneal lymphadenopathy), cisplatin could not be delayed because the patient suffered from a disseminated germ cell cancer and thus had a vital indication for immediate cisplatin initiation. Hence, a strategy of omitting uCrCl measurement and only measuring the eGFR would not have resulted in harm for any of the 121 patients in any of their 480 treatment cycles. We thus believe that the current data can reassure cancer centers that a simple determination of the eGFR is sufficient for kidney function assessment in patients undergoing cisplatin therapy. This is further supported by previous studies in cancer and non-cancer patients demonstrating that eGFR *estimation* equations are similarly accurate as GFR *measurement* strategies^30,31^.

Nonetheless, our results were generated in a cohort of patients who have received at least one cycle of cisplatin. As this was the selection criterion for defining the study cohort, our analysis cannot answer whether it may also be safe to omit uCrCl measurement at the time of cisplatin indication, because the decision on nephrologic eligibility for cisplatin therapy was made before the inclusion of patients into the present study. Based on this fact as well as the results of the current analysis, our institution has now adopted the policy of measuring both the eGFR and the uCrCl at the time of cisplatin indication (i.e. the first cycle for most patients), and then only measuring the eGFR for all subsequent cisplatin cycles. Otherwise, we speculate that the nephrologic safety of cisplatin therapy may be further improved by determining kidney function as part of a structured cisplatin eligibility evaluation at least once using an exogenous tracer “gold standard” method, such as plasma clearance of iothalamate or Tc-99 m-DTPA. This approach would have the advantage of an even more precise kidney function estimate before cisplatin therapy, but have the disadvantages of increased cost, limited global availability outside tertiary care facilities, patient burden, and risks associated with iodine exposure. Moreover, because these methods represent tubular excretion fraction, interpretation of test results may be difficult for most oncologists familiar with creatinine clearance.

Secondary endpoints of the study focused on the agreement between the eGFR and the uCrCl. For this analysis we used linear mixed models that take into account the hierarchical structure of our data, i.e. the fact that individual kidney function measurements are clustered within single patients^28^. Also, the Bland–Altman analysis of agreement was specifically adjusted for this data structure^24^. Here, we found that the overall agreement between the eGFR and uCrCl was relatively modest, and a non-negligible proportion of patients had highly outlying uCrCl measurements in both directions. These systematic results confirmed ad-hoc clinical observations in daily routine at our department and in the previous literature that many patients have compliance problems with 24-h urine collection^11^, resulting in erroneous input values for the uCrCl equation, sometimes resulting in uCrCl measurements in our cohort as low as 10 ml/min/1.73m^2^ or as high as 260 ml/min/1.73m^2^ in an otherwise nephrologically fit patient with a normal eGFR.

In a tertiary endpoint analysis, we looked at changes in kidney function during chemotherapy, again applying linear mixed models, which were recently advocated as the “gold standard” statistical technique for this type of data^28^. Interestingly, neither the eGFR nor the uCrCl declined during chemotherapy. Also, only 2 patients developed a relative eGFR decline ≥ 30% over time (an endpoint that is used in clinical trials for prospective kidney function impairment)^26^, and eGFR was above 50 ml/min/1.73m^2^ in these two patients even at the time of these declines. This supports the hypothesis that our relatively forced institutional cisplatin rehydration protocols (see Supplementary Table 1 for an example) in conjunction with stringent patient selection for cisplatin reduce the risk of cisplatin-induced acute kidney injury to negligible levels.

In a sensitivity analysis, we used age-, sex-, and race-specific thresholds from patients with CKD stages 1–5 to identify samples with potentially incomplete 24-h urine collection based on their ratio of 24-h urine creatinine to body weight^27^. Here, a large proportion of samples (i.e. roughly a third of all cycles) had potentially incomplete urine collection, although this has to be interpreted by considering that the respective thresholds for incompleteness were obtained from patients with early-stage CKD while our cohort predominantly consisted of patients without CKD. Importantly, our main finding prevailed upon excluding these samples, thus supporting our conclusion that assessing kidney function with an estimated GFR only is safe in patients with solid cancers undergoing cisplatin therapy who have received at least one cycle of cisplatin.

Finally, several limitations of the current study should be mentioned. First, the design of the study is retrospective, which always opens up the possibility of selection and information bias. We have addressed this possibility by clearly specified in- and exclusion criteria for generating a patient population that was treated at a single ward of a single academic cancer department with standardized cisplatin indication and rehydration protocols as well as creatinine determination in the same laboratory. Nonetheless, a prospective study design may have yielded a more robust answer to our study question. Second, eGFR was estimated with the CKD-EPI formula. Although this can be considered a strength, our data do not automatically generalize to settings where other eGFR estimation formulae, such as Modification of Diet in Renal Disease (MDRD), are used. Nonetheless, we can clearly advocate using the CKD-EPI formula for pre-cisplatin kidney function assessment rather than the MDRD formula, because MDRD was developed using data from patients with chronic kidney disease (CKD), has been shown to be less accurate in patients with an eGFR near or above 60 ml/min/1.73 m^2^ (i.e. the kidney function spectrum that is most relevant for cisplatin therapy), and is not as broadly validated across different clinical conditions and populations as the CKD-EPI formula^32–34^. Third, the focus of this study was cisplatin-induced acute kidney injury. Thus, other (albeit less serious) acute renal complications of cisplatin such as hypomagnesemia^35^, and hypokalemia were not considered. Fourth, the proportion of patients receiving very high-dose cisplatin therapy (single cisplatin doses of 100 mg/m^2^) was very low in our cohort. Thus, our results showing very low cisplatin toxicity may not be as low when studying a larger number of very high-dose cisplatin patients, such as those being treated with single-dose 100 mg/m^2^ cisplatin in definitive chemoradiation protocols for squamous cell carcinoma of the head and neck^36^. Fifth, other and potentially even more accurate forms of GFR measurement, such as the inulin clearance (no longer available) or iothalamate clearances^10^, were not investigated. However, as these methods are not routinely available outside of research or academic medicine settings, their relevance for our study question is negligible. Sixth, uCrCl was assessed by 24-h urine collection. As 24-h urine collection was done by patients independently at their home, it cannot be ruled out that some patients may have been incompliant or inexact with the 24-h collection period. Whether this was the case for individual patients cannot be ascertained within this retrospective study. Seventh, out conclusions could be biased by the fact that low muscle mass in association to tumor wasting and chemotherapy complications can lead to low creatinine concentrations in serum and urine and thus falsely high GFR values^27^. We believe that the magnitude of this bias in our analysis is likely very limited, because patients undergoing cisplatin therapy are selected for good performance status, do not have moderate or high CKD, and the CKD-EPI formula has shown high agreement with measured GFR (such as the no-longer available inulin clearance) in patient populations with a high prevalence of muscle wasting, such as patients after liver transplantation or patients with cirrhosis^37–39^. Also, weight as an imperfect proxy for muscle mass only declined by an average of 0.4 kg/cycle in our population which received a median number of 4 cycles. Finally, it is plausible that cisplatin may cause very late impairment of kidney function, in a similar fashion as it can result in ototoxicity and cardiovascular complications years to decades after cisplatin therapy^40^. Nonetheless, these potential late complications were not examined in the current study.

## Conclusion

We conclude that a strategy of assessing kidney function with an estimated GFR only (i.e. omitting direct GFR measurement with the uCrCl) is safe in patients with solid cancers undergoing cisplatin therapy who have received at least one cycle of cisplatin. This finding can help simplify renoprotective supportive care for the global population of patients requiring treatment with cisplatin by allowing patients and physicians to omit the burdensome timed urine collection before every cisplatin cycle.

## Supplementary information

Supplementary file1

## Data Availability

The dataset underlying this analysis can be shared upon reasonable request to the corresponding author (florian.posch@medunigraz.at).
