# Supplementary material for: Estimation versus measurement of the glomerular filtration rate for kidney function assessment in patients with cancer undergoing cisplatin-based chemotherapy
Source: Sci Rep. 2020 Jul 8;10:11219. doi: 10.1038/s41598-020-68010-5 (PMC7343883; doi:10.1038/s41598-020-68010-5)

Supplementary Data file

ESTIMATION VERSUS MEASUREMENT OF THE GLOMERULAR FILTRATION RATE FOR KIDNEY FUNCTION ASSESSMENT IN PATIENTS WITH CANCER UNDERGOING CISPLATIN-BASED CHEMOTHERAPY

Running Head: Kidney function assessment methods for cisplatin therapy

Marie-Christin Klöckl, MD[1] Anne-Katrin Kasparek, MD,[1] Jakob M. Riedl, MD,[1] Florian Moik, MD,[1] Stefanie Mollnar,[1] Michael Stotz, MD PhD,[1] Joanna Szkandera, MD,[1] Angelika Terbuch, MD,[1] Prof. Armin Gerger, MD MBA,[1,2,3] Tobias Niedrist, MD,[4] Prof. Martin Pichler, MD MSc,[1,5,6] Prof. Thomas Bauernhofer, MD,[1] Gernot Schilcher, MD,[7] Prof. Sabine Zitta, MD,[7] Prof. Alexander R. Rosenkranz, MD,[7] Claudia Friedl, MD,[7] Prof. Herbert Stöger, MD,[1] and Florian Posch, MD MSc[1,2]

[1]Division of Oncology; Department of Internal Medicine; Comprehensive Cancer Center Graz; Medical University of Graz; Graz, Austria

[2]Center for Biomarker Research in Medicine (CBmed); Graz, Austria

[3]Research Unit "Genetic Epidemiology and Pharmacogenetics"; Medical University of Graz; Graz, Austria

[4]Clinical Institute of Medical and Chemical Laboratory Diagnostics, Medical University of Graz; Graz, Austria

[5]Research Unit "Non-Coding RNAs and Genome Editing in Cancer"; Medical University of Graz; Graz, Austria

[6]Department of Experimental Therapeutics, University of Texas M.D. Anderson Cancer Center; Houston, TX, USA

[7]Division of Nephrology, Department of Internal Medicine; Medical University of Graz; Graz, Austria

Supplementary Tables

Supplementary Table 1. Rehydration and supportive care protocol for moderate-to-high-dose cisplatin chemotherapy regimens with a daily cisplatin dose between 40mg/m² and 80mg/m². The example below is for day 1 of Cisplatin(70)/Gemcitabine(1000), as used for (neo-)adjuvant therapy of muscle-invasive bladder cancer.

medication/chemotherapy	Dose/i.v. volume	period of time
pre-chemotherapy		
Glucose 5 %	1250 ml	
NaCl 0,9%	700 ml	
KCl	40 ml	over 2 hours (1000 ml/h)
Mg-Gluconicum 10%	40 ml	
Ca-Gluconicum 10%	50 ml	
Mannitol 15 %	250 ml	over 30 min
Dexamethasone	12 mg	in 100 ml NaCl 0,9% over 10 min
Granisetrone	3 mg	in 100 ml NaCl 0,9% over 10 min
Cisplatin	70 mg/m²	in 250 ml NaCl 0,9% over 30 min<br>+ 4A NaCl 10% (=40 ml) over 1 h
Gemzar	1000 mg/m²	in 250 ml NaCl 0,9% over 30 min
**post-chemotherapy**		
Glucose 5 %	1750 ml	
NaCl 0,9%	1000 ml	
KCl	60 ml	over 6 hours (500 ml/h)
Mg-Gluconicum 10%	40 ml	
Ca-Gluconicum 10%	60 ml	
Elomel isoton	1000 ml	8pm(day 1) - 8am(day2, 85 ml/h)

Day 8 (outpatient treatment) Gemzar	1000 mg/m^2	in 250 ml NaCl 0,9% over 30 min

Comment:

- 8 pm (day 0) -8 am (day 1): 1000 ml Elomel isotone (85 ml/h)
- Prophylaxis of gastritis with proton-pump inhibitor
- Laxatives if needed
- Aprepitant 125 mg on day 1, 80 mg on day 2 and 3
- Oral dexamethasone 4 mg 2x1 on day 2-4

Supplementary Figures

Supplementary Figure 1. Flow-chart of included patients and their GFR estimates and measurements included in the analysis.

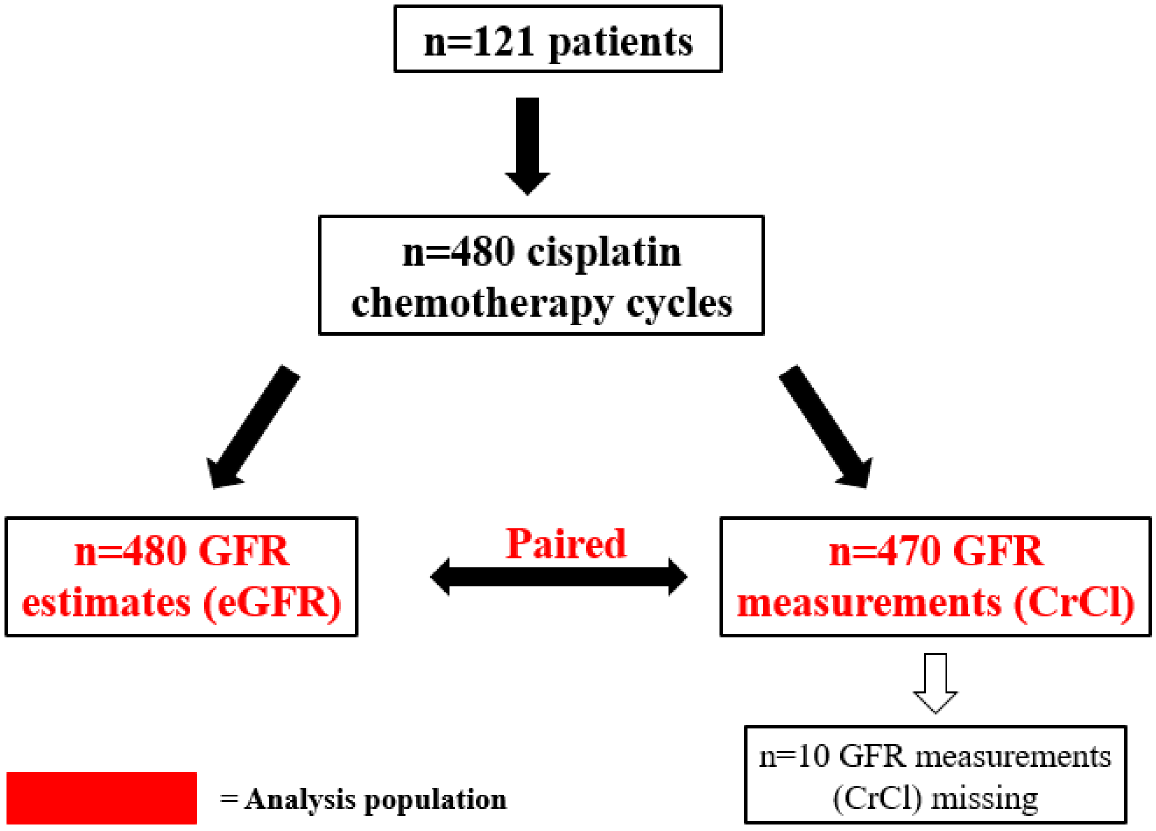

Supplement: Supplementary file 1 — Supplementary file1 [file 41598_2020_68010_MOESM1_ESM.pdf]
